# Editorial: The biology and management of chronic diseases in Mexican Americans

**DOI:** 10.3389/fmed.2022.1095203

**Published:** 2022-12-13

**Authors:** Eron Manusov, Belinda Reininger, Alfredo Ulloa-Aguirre, Sarah Williams-Blangero

**Affiliations:** ^1^Department of Human Genetics, University of Texas Rio Grande Valley-School of Medicine, Brownsville, TX, United States; ^2^University of Texas Health Science Center at Houston School of Public Health Brownsville Campus, Brownsville, TX, United States; ^3^Instituto Nacional de Ciencias Médicas y Nutrición Salvador Zubirán, National Autonomous University of Mexico, Mexico City, Mexico

**Keywords:** Mexican Americans, chronic illnesses, social determinants of health, community wide interventions, diabetes, frailty

Mexican Americans are at higher risk than the general population for the development and sequelae of chronic diseases, such as diabetes, hypertension, obesity, hyperlipidemia, and depression (1–4). The reasons are complex and lie in the interactions of genetics, health care, behavior, environmental factors and the social determinants of health (SDOH). The review topic discussed in this editorial comprises six papers focused on the Rio Grande Valley (RGV) region of South Texas where ninety percent of the population is of Mexican descent. The research papers cover emerging strategies for identifying and defining contributors to chronic disease, the epidemiology of chronic disease in Mexican Americans, the impact of population-based interventions for diagnosis and treatment, and the multiple, interacting factors that contribute to health and disease in Mexican Americans.

All six papers address the higher prevalence of chronic disease in the Mexican ancestry population of the RGV and the health disparities present in the region. The roles of adverse SDOH, poverty, and environmental stressors on risk for type to diabetes mellitus (T2D), obesity, hypertension, and depression are explored. Two of the papers report successful interventions associated with the implementation of culturally attuned and language congruent community-based programs designed for the Mexican American population.

The topic review includes data from the Mexican-ancestry population living on the U.S-Mexico border in the RGV. This population experiences high rates of complex diseases such as T2D (prevalence of 32%), obesity/overweight (60%), fatty liver disease (60%), and depression (19%) [Zhang et al.; (2, 5)].

Examining data from two HIV treatment clinics, Lopez-Alvarenga et al. detail the higher prevalence of T2D in people with HIV infection in the RGV. The increased prevalence is not associated with obesity, suggesting that factors independent of obesity may be partly responsible for T2D pathogenesis in HIV positive individuals. HIV positive individuals were more likely to develop T2D at younger ages than non-infected individuals independent of obesity status.

The paper by Garcia-Oropesa et al. provides a systematic review of randomized clinical trials of anti-obesity treatments in the Mexican-ancestry adult population, including studies in Mexico and US-Mexico collaborations. The trials included in the review covered alternative medicine, pharmacological, nutritional, behavioral, and surgical interventions and reported metabolically relevant traits such as BMI, weight, waist circumference, triglycerides, and glucose. The small sample sizes for many of the studies indicate the need for further larger-scale collaborative clinical trials focused on treatment of obesity.

Community-wide interventions were the focus of several papers in the topic review. The paper by Martinez et al. reviews what motivated participants who completed a free community-based weight loss program. The qualitative research approach used identified motivational themes based on the Self-Determination Theory and determined that external sources of motivation (physical appearance and financial remuneration) were more important than intrinsic motivations. However, the authors noted the need to create programs that emphasize intrinsic motivation, since they may be more effective for achieving long-lasting behavioral change.

Salud Y Vida (Reininger et al.) and Tu Salud ¡Si Cuenta! (Zhang et al.), are both culturally- and health literacy- attuned, multidisciplinary community-collaborative interventions. Both studies report on the benefits of the involvement of community health workers in public health interventions. Zhang et al. used Bayesian multilevel spatial models to investigate individual- and community-level SDOH related to diabetes management. The authors confirmed the roles of SDOH and lower SES in chronic illness. Zhang et al. went beyond reporting program outcomes and examined how different SDOH contribute to hemoglobin A1c (HbA1C) reductions following program engagement. Modifying factors included older age at baseline, being married (compared to being widowed or divorced), and English speaking (compared to Spanish), and were significantly associated with greater HbA1C reduction. Every percentile reduction in the SES advantage index corresponded to a.018 decrease in HbA1C. They found that community-level factors SDOH including socioeconomic advantage, urban core opportunity, immigration, and cohesion accessibility impacted risk for T2D, and suggested that these factors be considered when developing intervention programs.

Reininger et al. examined the dissemination and implementation of a model program, a community-wide campaign, to address obesity and hypertension in a region noted for its low SES and a high prevalence of chronic diseases. The authors utilized the evidence-based implementation framework (RE-AIM) to evaluate the Reach, Effectiveness, Adoption, Implementation, and Maintenance of the intervention. The results indicated that there is an association between program implementation and reduced blood pressure and reduced BMI in the RGV population. The intervention reached 12 community sites. The authors determined that exposure to the program, including follow-up visits, was important to success. The community-wide campaign was demonstrated to have acceptability for implementation in Hispanic populations and was associated with improved health outcomes.

Frailty is a measure of age-related decline in wellbeing. The effect of weight control, diabetes, hypertension, and depression on frailty in two local predominantly Mexican American communities was reported by Manusov et al. The authors calculated a ratio of suffered health deficits and total health deficits (Frailty Index-FI) for each of the communities. The FI can be used to determine the prevalence and trajectory of frailty. The FI comprises measures routinely measured (age, BMI, systolic/diastolic blood pressure, HbA1C, lipids (total cholesterol, high density, and low density and triglycerides), the Patient Health Questionnaire (PHQ9) and the Duke Profile Health-Related Quality of Life Questionnaire (physical health, mental health, social health, general health, perceived health, self-esteem, anxiety, anxiety/depression, and pain/disability). The authors found evidence for significant age and sex effects on FI, and found a significant difference in FI values between the two communities examined indicating localized variation in FI.

Overall, the review topic provided rich data on the health of an important US-Mexico border region. The authors contributing to this review topic describe health disparities in the region, including frailty, HIV infection, T2D, obesity, and other common chronic illnesses that are highly prevalent in the Hispanic population. Successful examples of health intervention programs demonstrate that high-quality, evidence-based public health interventions must include community involvement and interprofessional collaboration, and that they can be scaled up using an implementation framework.

Several essential questions for future consideration are raised by the papers included in the topic review. What components and strategies in community health initiatives are most effective for decreasing health disparities in vulnerable populations? How can intrinsic motivation for better health outcomes be best achieved in Hispanic populations? How do interventions modify health outcomes? How do genes, environmental factors, culture, immigration status, education level, SES, and other factors modify health and illness? Future directions should include collaborations in epidemiological research, implementation science guided interventions, community-based healthcare, and research strategies focused on vulnerable populations.

## Author contributions

EM and SW-B contributed to the topic concept, editorial efforts, and the development of the editorial. BR and AU-A contributed to the topic concept, editorial efforts, and editorial review. All authors contributed to the article and approved the submitted version.
